# Motivation and treatment engagement intervention trial (MotivaTe-IT): the effects of motivation feedback to clinicians on treatment engagement in patients with severe mental illness

**DOI:** 10.1186/1471-244X-12-209

**Published:** 2012-11-24

**Authors:** Eline C Jochems, Cornelis L Mulder, Arno van Dam, Hugo J Duivenvoorden, Sylvia CM Scheffer, Willem van der Spek, Christina M van der Feltz-Cornelis

**Affiliations:** 1 Department of Psychiatry, Epidemiological and Social Psychiatric Research institute, Erasmus University Medical Centre, Dr. Molewaterplein 50, Rotterdam, 3015 GE, The Netherlands; 2 GGZ Westelijk Noord Brabant, Post Office Box 371, Bergen op Zoom, 4600 AJ, The Netherlands; 3 2e De Carpentierstraat 232, The Hague, 2595 HN, The Netherlands; 4 GGZ Breburg, Post Office Box 770, Tilburg, 5000 AT, The Netherlands; 5 Faculty of Social Sciences, Tilburg University, Post Office Box 90153, Tilburg, 5000 LE, The Netherlands; 6 Netherlands Institute of Mental Health and Addiction (Trimbos Institute), PO Box 725, Utrecht, 3500 AS, The Netherlands

## Abstract

**Background:**

Treatment disengagement and non-completion poses a major problem for the successful treatment of patients with severe mental illness. Motivation for treatment has long been proposed as a major determinant of treatment engagement, but exact mechanisms remain unclear. This current study serves three purposes: 1) to determine whether a feedback intervention based on the patients’ motivation for treatment is effective at improving treatment engagement (TE) of severe mentally ill patients in outpatient psychiatric treatment, 2) to gather insight into motivational processes and possible mechanisms regarding treatment motivation (TM) and TE in this patient population and 3) to determine which of three theories of motivation is most plausible for the dynamics of TM and TE in this population.

**Methods/design:**

The Motivation and Treatment Engagement Intervention Trial (MotivaTe-IT) is a multi-center cluster randomized trial investigating the effectiveness of feedback generated by clinicians regarding their patients’ treatment motivation upon the patients’ TE. The primary outcome is the patients’ TE. Secondary outcomes are TM, psychosocial functioning and quality of life. Patients whose clinicians generate monthly motivation feedback (additional to treatment as usual) will be compared to patients who receive treatment as usual. An estimated 350 patients, aged 18 to 65 years, with psychotic disorders and/or severe personality disorders will be recruited from outpatient community mental health care. The randomization will be performed by a computerized randomization program, with an allocation ratio of 1:1 (team vs. team or clinician vs. clinician) and patients, but not clinicians, will be blind to treatment allocation at baseline assessment. Due to the nature of the trial, follow-up assessment can not be blinded.

**Discussion:**

The current study can provide important insights regarding motivational processes and the way in which motivation influences the treatment engagement and clinical outcomes. The identification of possible mechanisms through which changes in the outcomes occur, offers a tool for the development of more effective future interventions to improve TM and TE.

**Trial registration:**

Current Controlled Trials NTR2968

## Background

Disengagement and non-completion of treatment pose a major problem for the successful treatment of patients with severe mental illness, since it is associated with several clinical and socio-economical problems such as recurrent psychiatric problems, rehospitalisation, and increased risk of suicide and episodes of violence [1-3]. Estimates of treatment disengagement vary across different psychiatric patient populations and depend on the definitions of disengagement and non-completion. For example, non-adherence to antipsychotic medication among patients with psychotic disorders was observed in over 50% of patients [4,5], while non-completion of personality disorder treatment is estimated at 37% [6]. Lehner et al. [3] found that among individuals in treatment for severe mental illness, appointment failures ranged from 50% to 73%, drop-out estimates ranged from 14% to 92% and medication failure estimates ranged from 5% to 71%.

Research into the determinants of treatment engagement and completion of treatment in severe mental illness has revealed numerous important factors, including patient-related factors (e.g. age, ethnicity, beliefs about treatment efficacy, income level, psychiatric history), illness-related factors (e.g. the type of disorder, symptom severity, comorbidity) and treatment-related factors (e.g. treatment setting, type of treatment, treatment efficacy, adverse treatment effects, therapeutic alliance) [2,7-9]. Although some of these factors are static and can not be influenced, others are more dynamic and may therefore be targeted in interventions to enhance treatment engagement. One dynamic factor that has long been recognized as an important determinant of treatment engagement is the patient’s motivation to make the efforts required by the treatment [9-12]. However, due to an abundance of conceptualizations of the global term ‘motivation’, it has proven difficult for both academics and clinicians to effectively work with the concept. As Drieschner et al. [12] point out, despite a large amount of publications regarding treatment motivation, the concept remains ill-defined and is therefore a continued source of confusion. Therefore, more insight into the associations between determinants of motivation, actual motivation to engage in treatment, treatment engagement and psychosocial functioning may contribute to the effectiveness of psychiatric interventions.

This article describes the study protocol for the Motivation and Treatment Engagement Intervention Trial (MotivaTe-IT). MotivaTe-IT serves two purposes: 1) to determine whether a feedback intervention based on the patients’ motivation for treatment is effective at improving treatment engagement (TE) of severe mentally ill patients in outpatient psychiatric treatment, and 2) to gather insight into motivational processes and possible mechanisms regarding motivation for treatment and treatment engagement in this patient population. In the following, we will describe why we chose to use motivation feedback as the intervention in this study.

### Motivation feedback intervention

Studies employing feedback to clinicians have shown that monitoring and informing clinicians of their patients’ treatment progress in psychotherapy is effective in enhancing retention and outcome [13-18]. Providing systematic feedback can be seen as an addition to regular treatment and may guide changes, prolongation or termination of treatment. It ensures that the attempts to resolve the problems can be evaluated, and if necessary, adjusted [19]. In several studies by Lambert et al. [13,14,16] in a psychotherapy setting, progress feedback was based upon four domains of functioning, including psychological disturbance (mainly depression and anxiety), interpersonal problems, social role functioning and quality of life [17]. The effects of feedback were most pronounced in patients who showed a poor initial response to treatment [15]. Feedback is also increasingly being researched in other settings. In a study in patients with psychotic disorders in a community mental health setting, patients were asked to rate their quality of life and satisfaction with treatment, which was fed back to clinicians and discussed [20].When compared to control patients (who did not make use of feedback) after 12 months, patients in the feedback condition reported better quality of life, fewer unmet care needs and higher satisfaction with treatment. However, the groups showed no statistically significant difference on psychopathology scores (i.e. positive, negative or general symptoms of schizophrenia). In another study conducted among SMI patients receiving community care, where clinicians received feedback on their patients’ care needs, a significant improvement was found in patient satisfaction, but not on psychopathology, social functioning and quality of life [21] when compared to controls. A study conducted in the Netherlands among patients with severe mental illness, found that systematic monitoring of patients’ care needs in combination with feedback provision was associated with global improvement in depression and anxiety symptoms, but not with improvement in manic excitement and positive symptoms [22]. It seems that structured feedback has positive effects on some central outcomes of community mental health care (e.g. quality of life and patient satisfaction) but not on others (e.g. level of symptoms or functioning), depending on the setting and the content of the feedback.

In a study by Whipple et al. [16] a more extensive form of feedback was used when compared to the Lambert et al. studies [13,14], where the authors found that using clinical support tools (CSTs) additional to feedback upon the client’s progress resulted in clients staying in therapy longer, and that these clients were twice as likely to show superior outcomes. These CSTs incorporated measures to assess the therapeutic relationship, the motivation to change and the social support network. These results line up with other studies about feedback to clinicians and point out that the use of support tools is of additional value [16]. However, a limitation of Whipple’s study was that it was not possible to determine the effects of the individual components (e.g. motivation to change) in the CSTs upon outcome. Some studies have compared the effects of personalized feedback with the effects of motivational interviewing including personalized feedback, and found that feedback only is less effective than motivational interviewing with feedback in achieving behaviour change [23-25]. Therefore, next to providing feedback, it seems important to apply additional strategies in order to improve the motivation of patients to engage in treatment.

The aforementioned clinician feedback research has focused primarily upon treatment progress and was unable to determine which specific element(s) from the clinical support tools provided the mechanism(s) of action. Since treatment motivation has been found to be of crucial importance in this matter [9-12], the current study set out to place treatment motivation in a central position. The feedback that will be provided to the clinicians in the current study revolves around the patients’ motivation to engage in their treatment. Therefore, our feedback intervention is labelled motivation feedback. The feedback to clinicians will be based upon the current motivational state of their patients regarding their motivation for remaining and engaging in treatment.

Furthermore, solely providing feedback to clinicians of patients with severe mental illness might not be sufficiently intensive to improve treatment engagement [16]. To aid clinicians in addressing motivational problems that become evident from the feedback, clinicians will be educated in motivation enhancement strategies based on Self-Determination Theory [26,27]. Despite the differences between the Transtheoretical Model [28], the Integral Model of Treatment Motivation [12], and Self-Determination Theory [26] on the concept of treatment motivation, these theories may complement each other [29]. A detailed discussion of similarities and differences in how these three theories predict treatment engagement and outcomes can be found in Jochems et al. [29].

We chose Self-Determination Theory (SDT) [27] as the basis of our motivation feedback intervention, since this theory encompasses both a qualitative and quantitative view of motivation and the intervention strategy that it implies seems suitable for patients with SMI. In brief, SDT postulates different types of motivation, where the most central distinction is made between autonomous (i.e. self-determined) motivation and controlled (i.e. externally determined) motivation. Autonomous motivation may vary from intrinsic motivation to types of extrinsic motivation in which people have identified with the value of a change and have integrated this change into their sense of self [30]. SDT poses that autonomously motivated people experience greater ownership of the behaviour, will have greater intention to persist in treatment and have better mental health outcomes [30,31]. In contrast, controlled motivation consists of external regulation, in which behaviour is regulated by external rewards or punishments, and introjected regulation, where the drive for behaviour is partially internalised and energised by avoidance of shame, guilt and anxiety [27]. When people have a controlled motivation, they will show poorer health outcomes according to theory [30]. Furthermore, SDT states that fulfilling the patients’ basic psychological needs of autonomy, competence and relatedness during treatment will facilitate internalization of motivation for treatment, leading to better health outcomes [30].

## Methods/design

### Aims

The study has three main objectives. The primary objective is to determine the effects of the motivation feedback intervention on treatment engagement (TE) of patients with psychotic and/or personality disorders. Secondary outcomes are the patient’s treatment motivation, psychosocial functioning and quality of life. To this end, clinicians will be randomly assigned to either of two groups; one group will generate SDT-based feedback on the motivation of their patients while the other group will not.

The second objective is to determine the factors associated with the effect of our motivation feedback intervention upon the primary and secondary outcomes. Several demographic and clinical factors as well as factors that have a theory-based and/or empirically established relationship with the outcomes will therefore be assessed. At the moment, it is unclear which exact factors are most important so this will be studied explorative.

The third and final objective of the study is to determine which theory of motivation is most plausible for the dynamics of TE and treatment motivation in patients with psychotic disorders and personality disorders in outpatient treatment. The models selected here are the Transtheoretical Model (TTM) [28], the Integral model of treatment motivation (IM) [12] and Self-Determination Theory (SDT) [27]. In a literature review that we have performed earlier, we have described these theories in detail, including their differences and similarities [29]. We will explore which of three theories (i.e. TTM, SDT, and IM) is most supported by the data in predicting treatment motivation and engagement. It is possible that different subcomponents of these theories will be integrated in a novel theoretical-empirical model tailored to this specific population.

### Hypotheses

**Figure 1:**
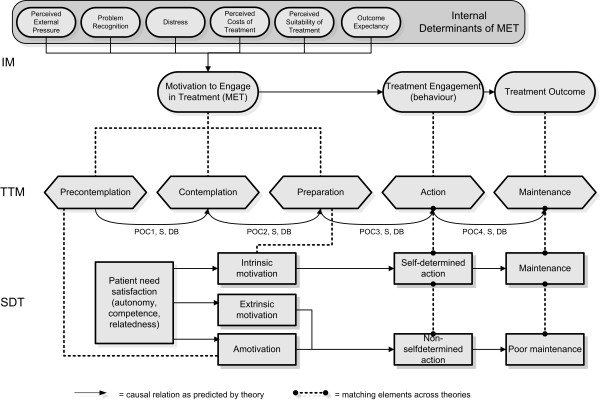
**Visualization of the three motivation theories and their interrelations. IM**: Integral Model; **TTM**: TransTheoretical Model; POC1: Processes of change (consciousness raising, dramatic relief); POC2: Processes of change (self-reevaluation); POC3: Processes of change (self-liberation); POC4: Processes of change (reinforcement management, helping relationships, counterconditioning, stimulus control); S: Self-efficacy; DB: Decisional Balance; **SDT**: Self Determination Theory.

It is hypothesized that motivation feedback to clinicians on the treatment motivation of their patients will lead to an increase in both the quantity and quality of treatment motivation and treatment engagement of these patients. The patient’s self-reported motivation and the clinician-reported motivation of the patient are expected to induce more awareness regarding motivational issues that are at play during treatment, and subsequently to more suitable (motivational) interventions leading to better outcomes (i.e. treatment engagement and psychosocial functioning). More specifically, we expect the increase in quantity and quality of motivation will follow the patterns shown in Figure 1. For example, in the intervention group we expect a larger increase in autonomous motivation (concept from SDT), a larger proportion of patients making forward shifts in the stages of change (concept from TTM) and a larger increase in motivation to engage in treatment (concept from IM) relative to the control group. As a consequence, we expect the intervention group to show a higher level of treatment engagement than the control group at the time of follow-up, as demonstrated by higher clinician-rated treatment engagement, less no-shows and better antipsychotic medication adherence in the patients with psychotic disorders.

### Treatment groups

#### Control condition: treatment as usual

The control condition consists of patients who are provided treatment as usual (TAU). These patients receive care that is guided by their individual symptoms, problems and needs. Treatment may consist of assertive outreach, medication, cognitive (behavioural) therapy, stress-management, family therapy, and/or supportive structured therapy. Assertive outreach is provided by Flexible Assertive Community Treatment (FACT) teams. FACT is a team treatment model that aims to provide community-based, assertive, outreaching and supportive psychiatric services to individuals with SMI [32,33]. Besides assertive outreach, which is the key feature of Assertive Community Treatment (ACT), there is an emphasis on out-of-office interventions and home visits, but when patients constitute a danger to themselves or others and are not motivated for treatment, clinicians can start a procedure for them to be committed to a psychiatric hospital [33]. During hospitalisations, the ACT team keeps into contact with the patient to secure continuity of care. In the Netherlands, a special type of ACT teams exist, called Flexible-ACT (FACT). Van Veldhuizen (2007) has described Dutch FACT as follows: “FACT is a rehabilitation-oriented clinical case management model, which is based on the ACT model but is more flexible and able to serve a broader range of clients with severe mental illness. FACT offers the original ACT as one of several treatment or care models. The FACT team is a case management team with partly an individual approach and partly a team approach; the approach varies from patient to patient, depending on the patient’s needs. For more stable long-term patients FACT provides coordinated multidisciplinary treatment and care by individual case management. Unstable patients at risk of relapse, neglect and readmission are provided with intensive assertive outreach care by the same team, working with a shared caseload for this subgroup. (p.422)” Patients and clinicians in the TAU condition will be assessed at baseline and at 12 months follow-up. Type, duration and frequency of TAU will be monitored.

### Intervention condition: motivation feedback

**Figure 2:**
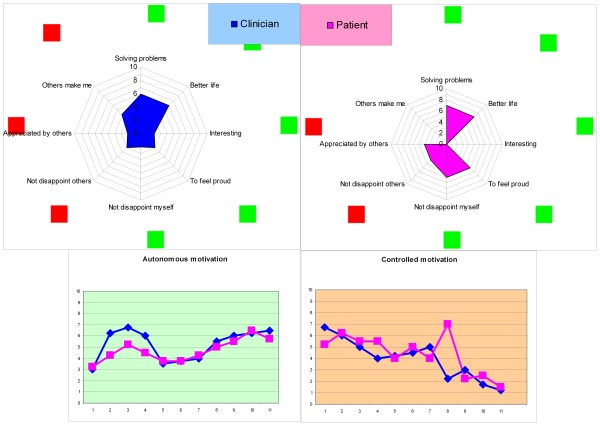
**Hypothetical motivation feedback: the motivation profile by the clinician and patient (top parts) and the course of motivation (bottom graphs).** The top part shows that although the clinician and patient agree that the patient is currently in treatment to solve problems and aim for a better life, the patient indicates that he also finds it important to feel proud of himself and to not disappoint himself. This could be a starting point for the discussion. In the lower graphs, it can be seen that the autonomous motivation had risen in the first three measurements and then dropped in the subsequent two measurements, at which point the clinician might choose to intervene.

Patients randomized to the motivation feedback condition will receive treatment as usual (TAU) and additionally, their clinicians will generate information regarding the patient’s motivation to engage in treatment. Patients and clinicians in the intervention group will fill in a short motivation feedback questionnaire every month up to twelve months after baseline assessment that provides the clinicians with motivation feedback. The short motivation feedback questionnaire includes eight statements that relate to the level and type of the patient’s treatment motivation, based on two types of motivation as distinguished by SDT. The individual items of both clinician and patients are rated on a 10-point continuous scale and can be plotted against each other in a graph to represent visual motivation feedback to the clinician. This graph then shows both the patient’s rating and the clinician’s rating of the current level of autonomous and controlled motivation of the patient. Figure 2 presents a hypothetical motivation profile and graphs of a possible course of the motivation over time.

Previous pilot testing with the short motivation feedback questionnaire among 55 patients with primarily anxiety and depressive symptoms receiving outpatient treatment showed that the list was comprehensible and easy to use in clinical practice. Clinicians appreciated the brevity and clarity of the items, which could function as a starting point for the discussion with the patient regarding his/her current motivation to engage in treatment.

Clinicians will be asked to fill in the short motivation feedback questionnaire just before the appointment with the patient. After having filled in the questions, the clinician will ask the patient at the beginning of the appointment to also fill in the questions on motivation for treatment. This information will be used by the clinician as a starting point for the discussion with the patient regarding his/her motivation for treatment. Clinicians randomized to the feedback condition, are expected to measure and discuss the current motivational status of their patients monthly. The clinician may use the information from the questionnaire and the subsequent conversation with the patient about this as feedback and apply an intervention tailored to the patients’ current motivation. Clinicians will be free to decide for themselves how they will structure this discussion with the patient (e.g. discuss only one item or several, discuss differences between patient and clinician vision) and how long this will take. In case the patient is unable or unwilling to indicate his/her motivation, the clinician may still use his own judgment of the motivation of the patient and use this as self-generated feedback. Additionally, the motivation of the clinician to keep treating the particular patient is also measured monthly by asking the clinician to rate two other motivation items.

Before commencing the study, clinicians will be trained by the principal investigator how to read and interpret the motivation feedback graphs. During this training, they are given a presentation about the principles of Self Determination Theory, the different types of motivation postulated by SDT and perform exercises to learn how to distinguish the needs for autonomy, competence and relatedness in discussions with the patient. Clinicians also perform feedback assessments on each other during this training, to familiarize themselves with the feedback and how to introduce it to their patients. During the course of the study (i.e. one year) clinicians will be regularly contacted by the principal investigator to evaluate the motivation feedback intervention and to discuss their progress and experiences together with other colleagues who also participate in the motivation feedback intervention. During the evaluation sessions with the principal investigator, it can be checked whether the feedback is still being used properly (and if not, actions can be taken). To aid clinicians in remembering to perform SMFL assessments monthly, they will be given MotivaTe-IT bookmarks to use in their paper planners, electronic reminders will regularly be placed in the electronic planners, and the principal investigator will send emails to remind the clinicians of the motivation feedback.

In case a patient is transferred to another clinician during the course of the study (e.g. in case of treatment by a FACT-team where several clinicians cooperate to provide services to patients), the feedback generated by the patient will be provided to the clinician who is currently the primary clinician (i.e. case-manager) involved with the patient. The feedback generated by clinicians who have been engaged with the patient at an earlier moment in time will be provided to the clinician who is currently the primary clinician, so that it remains possible to keep monitoring the development of the patient’s motivation over time.

### Development of the motivation feedback intervention

The guidance provided by the UK’s Medical Research Council on developing and evaluating complex interventions (http://www.mrc.ac.uk/complexinterventionsguidance) states that the identification of evidence base and theory, the modelling of process and outcomes, assessing feasibility and piloting methods are important steps towards successful evaluations of complex interventions. The motivation feedback intervention under study here, although new in it’s emphasis on motivation for treatment as the content of feedback (as opposed to care needs or quality of life), is otherwise fairly similar to previously trialled clinician feedback where it was found that feedback improved SMI patient outcomes in community mental health settings [20-22]. As Self-Determination Theory is the theoretical basis for the intervention, this ensures that the effects (or potentially no effects) of the intervention can be viewed in light of the processes of change proposed by this theory. Pilot testing with the novel short motivation feedback questionnaire in a group of patients with depressive and anxiety disorders showed that the list was comprehensible and easy to use, for both patients and clinicians. The clinicians reported that the questionnaire gave rise to interesting discussions with patients about drives and motivations that the clinician was unaware of, such as partners or children being more important drives to remain in treatment than levels of distress, or patients expressing that they felt very much coerced to enter treatment at first (sometimes even traumatic) but felt that this had progressed to more internal drives during the course of treatment. These pilot evaluations strengthened our belief that the intervention could be executed as intended. Due to time limitations however, no piloting was done with patients with SMI and the psychometric properties of this questionnaire remain to be determined. These issues will therefore be addressed during the course of the trial.

### Design and setting

This study is a multicenter randomized controlled study with two treatment conditions: treatment as usual (TAU) and motivation feedback (additional to TAU). There will be two extensive measurement occasions for both groups: at baseline and follow-up at 12 months.

Twelve departments within the Mental Health Center West North Brabant (MHC WNB), and the Mental Health Center BreBurg (MHC Breburg) located in the south west of the Netherlands, were approached to participate in the study. The MHC WNB and MHC Breburg provide mental health care to varying patient populations, including patients with a primary diagnosis of psychotic and/or personality disorder who will be targeted for this study. The current study will take place at several treatment locations of the MHC WNB and MHC Breburg, and represents a partnership between these centers and the Epidemiological and Social Psychiatric Research institute (a research center within the Erasmus Medical Center in Rotterdam, the Netherlands).

### Study population: inclusion and exclusion criteria

The current study aimed for patients with severe mental illness treated in outpatient community mental health care, and although there are several definitions of severe mental illness, most definitions include a diagnosis of severe psychiatric disorder, a treatment duration or illness duration of at least two years and several disabilities [34,35]. Since patients with psychotic disorders constitute the majority of patients treated in assertive community mental health teams in the Netherlands [33,36] and patients with severe personality disorders constitute another significant part of the caseload, combined with clinical observations that these two diagnostic groups may especially benefit from interventions aimed at improving treatment motivation and treatment engagement, it was decided to incorporate both patient groups into the study.

The research participants will consist of patients with a primary diagnosis of a psychotic disorder and/or a personality disorder, and their clinicians. Patients are eligible for participation if they are aged between 18 and 65 years old and receive individual outpatient treatment for their psychotic and/or personality disorder. Exclusion criteria are insufficient command of the Dutch language and/or a documented diagnosis of organic psychosyndrome (e.g. dementia or chronic toxic encephalopathy).

Clinicians will be eligible for participation if they are the primary health care practitioner involved with the patient, meaning that he/she is the one that has the most frequent contacts with this patient. It is expected that the resulting group of clinicians will mainly consist of specialized social workers, specialized psychiatric nurses and psychologists with relevant treatment experience with this patient population.

### Methods

In order to test the three motivational theories while also trying to limit the level of response burden for study participants in our intervention trial, proper choices for instruments had to be made. To ensure that we measure constructs appropriately for each theory, we tried to stay as close as possible to the original measures used by Ryan and Deci [27] for SDT, Prochaska and DiClemente [37] for TTM and Drieschner et al.[38] for IM. Priority was given to readily available Dutch versions of measurement instruments, but in case these were not available we chose to apply a translation procedure to the original English versions. Since our motivation feedback intervention is based on SDT, the primary outcome analysis is focused on this theory. Subsequently we will investigate how well the other two theories explain the effects of the intervention. Table 1 gives an overview of the instruments – questionnaires and interviews – that will be applied at baseline, monthly (for the intervention condition only) and at 12 months follow-up to patients and clinicians. It is estimated that the total duration of the assessment for clinicians takes 25 minutes per measurement occasion, while for patients this is 70 minutes.

**Table 1:** Instruments used at two research contacts and monthly

	**Patients**		
	**T0 (Baseline)**	**Monthly**	**T1 (12 months)**
TMS-f	x		x
URICA-D	x		x
SoC Algorithm	x		x
PCS			x
TEQ	x		x
HCCQ	x		x
IS	x		x
Zoo Map test	x		x
HAQ	x		x
TCI	x		
MMAS	x		x
Stigma Scale	x		x
HoNOS*	x		x
BPRS*	x		x
MANSA*	x		x
SDT graph**		x	
	**Therapists**		
TMS-f	x		x
URICA-D	x		x
SoC Algorithm	x		x
HAQ	x		x
SES	x		x
SDT graph**		x	
Therapist motivation***		x	

* These measures are part of ROM as standard clinical practice, and are thus of no additional burden to the patient.

** Only patients and therapists in the motivation feedback condition fill in the SDT graph.

*** Only therapists in the motivation feedback condition fill in two items regarding their motivation to treat the patient.

**TMS-f:** Treatment Motivation Scale for forensic patients, **URICA-D:** University of Rhode Island Change Assessment – Dutch version, **SoC algorithm:** Stages of Change algorithm, **PCS:** Processes of Change Scale, **TEQ:** Treatment Entry Questionnaire, **HCCQ:** Health Care Climate Questionnaire, **IS:** Insight Scale, **HAQ:** Helping Alliance Questionnaire, **TCI:** Temperament and Character Inventory, **MMAS:** Morisky Medication Adherence Scale, **HoNOS:** Health of the Nations Outcome Scale, **BPRS:** Brief Psychiatric Rating Scale, **MANSA:** Manchester Short Assessment of Quality of Life, **SDT graph:** Self-Determination graph, **SES:** Service Engagement Scale.

### Primary and secondary outcomes

The primary outcome in this study is actual treatment engagement, as measured with the Service Engagement Scale (see paragraph on treatment engagement). Secondary outcomes in this study are treatment motivation, as measured with the Treatment Entry Questionnaire (see paragraph on SDT instruments), administrative data on missed appointments (see paragraph on treatment engagement), psychosocial functioning and quality of life (see paragraph on secondary outcomes).

#### Treatment engagement

Treatment engagement will be measured with the Service Engagement Scale (SES) that was constructed by Tait, Birchwood & Trower [39]. The SES has 14 items that are rated on a 4-point scale ranging from 0 (not at all) to 3 (most of the time). The four subscales refer to availability, collaboration, help seeking and treatment engagement. The scale will be administered to clinicians. The original English version of the SES has shown good psychometric properties [39]. As a more objective measure of treatment engagement, data from the patients’ files will be collected on the frequency of missed appointments with the main clinician, percentage of missed appointments over all appointments in the past year, reasons for discontinuation of care or drop-out (if applicable) and the number of admissions in the past year (voluntary and involuntary).

Furthermore, the Morisky Medication Adherence Scale (MMAS) [40] will be administered to only to patients with psychotic disorders to measure the level of antipsychotic medication adherence. The MMAS is a self-report scale that consists of 8 items asking about a specific medication-taking behaviour. The total scale score can range from 0 to 8, which will be discretized into high adherence (score of 8), medium adherence (score of 6 or 7) or low adherence (score below 6) [40]. The scale was found reliable (Cronbach’s α= 0.83) as a measure for blood pressure medication adherence in patients with hypertension [40] and has been adjusted to fit our study population of psychotic patients. Additionally, the psychiatrists of the patients with psychotic disorders will be asked every six months to indicate whether they believe the patient adheres to the antipsychotic medication and if not, to give reasons for the patient’s nonadherence.

#### SDT instruments

The types of motivation that are distinguished by SDT will be measured with the Treatment Entry Questionnaire (TEQ) [10,41]. It was shown that the TEQ was reliable (i.e. internally consistent) for external (Cronbach’s α = .89), introjected (Cronbach’s α = .89) and identified motivation (Cronbach’s α = .85) [41]. To our knowledge, the TEQ has not been studied in a Dutch population before. Therefore, we translated the original TEQ by Wild et al. [41] and adapted the wording to fit a population of patients with severe mental illness in psychiatric treatment (e.g. words that referred specifically to addiction treatment were replaced by words that reflected more general treatment by a mental health center). Two translators performed independent forward translations of the original TEQ into Dutch and adapted the wording to fit its application to outpatient psychiatric treatment. A consensus version was established, consisting of 27 items that can be rated on a 7-point Likert-scale ranging from 1 (strongly disagree) to 7 (strongly agree). The psychometric properties of this Dutch TEQ are to be investigated in this study.

The Health Care Climate Questionnaire (HCCQ) will be used to measure the degree to which clinicians are perceived to be autonomy supportive. Items are scored on a Likert scale, ranging from 1 (strongly disagree) to 7 (strongly agree). The HCCQ has 15 items that have been used in studies of weight loss [42] (Cronbach’s α =.92) and smoking cessation [43] (Cronbach’s α = .96). Application of a Dutch HCCQ is not known to us. Therefore, the original HCCQ was translated into Dutch by two independent translators who subsequently established a consensus version. This consensus version was back translated into English by two independent expert translators to check for discrepancies between the original version and the backtranslation. On the basis of consensus between all translators, the final Dutch questionnaire was achieved. The psychometric properties of the Dutch HCCQ will be determined in this study.

#### TTM instruments

The stages of change will be measured by staging algorithms and the University of Rhode Island Change Assessment – Dutch version (URICA-D). Algorithms are capable of placing individuals in one of five stages and have been used extensively in diverse populations and research areas [37,44,45]. The algorithm approach involves several questions that ask about attempts and intentions to change behaviour within certain time frames corresponding to a particular stage. Both patients and clinicians will be asked to judge whether the patient is currently in the precontemplation, contemplation, preparation, action or maintenance stage with regard to the patients’ motivation to change his psychiatric problems and specific problem behaviours if relevant (e.g. alcohol abuse, drug abuse and criminal behaviours). Precontemplation is defined as ‘not planning to work on my problems in the next six months’. Contemplation is defined as ‘planning to work on my problems within the next six months, but not within 30 days from now’. Preparation is defined as ‘planning to work actively on my problems within the next 30 days’. Action is defined as ‘having worked on my problems actively for the last 30 days, but no longer than six months’. Maintenance is defined as ‘having worked actively on my problems for the last six months’. These definitions are similar to other stage algorithms from TTM [46].

The URICA-D is the Dutch version of the URICA [47], which is a self-report scale that asks the patient to first enter a problem and then to indicate on a five point Likert scale (1 = strongly disagree to 5 = strongly agree) how much he agrees with a particular statement. The URICA-D consists of four subscales which represent four stages of change: precontemplation, contemplation, action and maintenance. The reliabilities (i.e. Cronbach’s alpha) for the subscales have been found to range from 0.84 to 0.95 [48].

The processes of change will be measured by asking patients to indicate how often they make use of the strategies described in 20 statements, where each process of change is represented by two statements. The statements are rated on a five point Likert scale, ranging from 1 (never) to 5 (repeatedly), consistent with other measures of the processes of change in TTM [49-51]. Application of the processes of change scale in a Dutch psychiatric patient population is not known to us. Therefore, we developed a questionnaire based on the original English questionnaire by Prochaska et al. [50] and adapted it to a population of people with mental illness in psychiatric treatment. Two translators performed independent forward translations of the Processes of Change Scale (PCS) [50] into Dutch and adapted the wording to fit its application to change processes in psychiatric treatment. From the 40 items generated in this translation procedure, a consensus version was established from which 20 items were chosen (two items per process) as most relevant to create a short form of the processes of change inventory, consistent with other short forms of the processes of change inventory (e.g. in the studies of [49,52]). The psychometric properties of our scale are to be investigated in this study.

The decisional balance constructs and self-efficacy constructs are incorporated in the Treatment Motivation Scale for forensic patients [38], a scale that will be used to measure the constructs of the IM (see next section).

#### IM instruments

The constructs within the IM will be measured by the Treatment Motivation Scale for forensic patients (TMS-f) [38]. The TMS-f consists of eight subscales, one scale for the motivation to engage in treatment (MET) and six scales for variables that are summarized as Internal Determinants of MET: problem recognition, distress, perceived legal pressure, perceived costs of treatment, perceived suitability of treatment and outcome expectancy. An additional scale assesses the patients’ tendency to respond according to social desirability. The items within the scale of ‘perceived legal pressure’ were adapted to fit a more broadly defined concept of perceived External Pressure, in order to fit all patients in our research population.

The TMS-f has a patient version (85 items) and a clinician version (7 items), and both will be used in our study. The TMS-f has been found to be a reliable and valid operationalisation of the constructs in IM [38,53,54]. However, the TMS-f has only been used in a forensic psychiatric setting and it remains to be determined whether the scale is also applicable outside this setting. In the total patient population in which the scale was validated, it was found that 61% of the patients had axis-I disorders, while strong characteristics of personality disorders were prevalent in 78% of patients [53,55]. The composite reliability of the scale ranges between α = .83 and α = .91 [38].

#### Psychosocial functioning

Psychosocial functioning will be measured with the Dutch version of the Health of the Nations Outcome Scales (HoNOS) [56,57]. The HoNOS form is completed via a semi-structured interview with the patient. The HoNOS quantifies health and social problems during the previous two weeks and contains 12 items that refer to behavioural problems, impairment, symptoms, alcohol and drug abuse, and social (dis)functioning. Three HoNOS addendum items are also administered. These refer to manic symptoms, treatment motivation and compliance with medication. The items are rated from 0 (no problem) to 4 (very severe problem). The HoNOS has shown to be reliable and sensitive to change [57]. In order to obtain a more differentiated understanding of the psychotic symptoms, five items from the Brief Psychiatric Rating Scale [58] will be administered additionally to the HoNOS items in the interview with the patient. These include suspiciousness, unusual thought content, grandiosity, hallucinations and blunted affect. The BPRS has been used in various settings and has shown good psychometric properties [59].

#### Quality of life

The Manchester Short Assessment of Quality of Life (MANSA) [60] will be used to measure quality of life. The MANSA is a self-report questionnaire administered to the patient to measure how satisfied the patient is in the following life domains: living situation, social relationships, physical health, mental health, safety, financial situation, work situation and life as a whole. Each question is answered on a 7-point scale (1 = not satisfied, 7 = very satisfied) and a composite (mean) score is calculated. The psychometric properties are satisfactory [60], and the scale has also been validated in a population of patients with severe mental illness [61].

### Covariables

#### Socio- demographic factors of patients and clinicians

Socio-demographic data on gender, age, ethnicity, marital status, living situation, housing, distance from the treatment location, educational background, income, treatment history, treatment duration, no-shows in the treatment in the previous twelve months, legal status, medication use, and DSM-IV diagnosis will be collected at baseline from the patient’s medical record. In case of missing information in the medical record, the patient will be asked to provide the information. Information on clinician sex, age, years of clinical working experience, and treatment team was collected from clinicians.

#### Insight into illness

Impaired insight has been associated with reduced treatment engagement and increased symptoms, as well as higher rates of involuntary detention [62]. The Insight Scale [63] will be used to measure a patients’ insight into illness. This 8-item self-report scale produces a total score that ranges between 0 and 12. It was found to be a reliable, valid and easily applicable measure [63].

#### Executive functions

There is considerable evidence for cognitive dysfunctioning, especially impaired executive functioning, in patients with severe mental illness [64-66]. Executive dysfunctioning has been found to contribute to poor insight in psychosis and might be related to poor treatment engagement [66]. As a measure for executive functions, planning ability was chosen. Although the Wisconsin Card Sorting Test (WCST; [67]) is typically administered as a measure for executive functioning [66], the inclusion of this test to our study instruments would increase the burden to the patients such that we decided it was unsuitable for administration. Alternatively, planning ability will be measured with the Zoo Map test, a subtest of the Behavioural Assessment of Executive Functioning (BADS) [68,69]. The Zoo Map test asks the patient to draw a route on a map of a zoo and to visit specific sites in the zoo while applying specific rules (e.g. ‘you can use the dotted pathways as often as you want, but the white pathways only once’). There are two subtests within the Zoo Map test: the first is unstructured, forcing the patient to plan his route independently. This indicates the extent to which the patient is capable of spontaneous planning. The second condition is structured and indicates a specific order in which the patient should visit the specific sites. This indicates the ability of a patient to follow a concrete, externally demanded strategy. Theoretically, it is expected that patients who find it difficult to develop logical strategies on the Zoo map test also have more difficulties with following a (complex) treatment regimen. The time used for planning and execution of the task and the number of mistakes (breaking a rule) are scored, and a profile score ranging from 0 to 4 for each subtest is then derived. The BADS has shown adequate validity and test-retest stability [69,70].

#### Therapeutic alliance

The therapeutic relationship is measured with the Helping Alliance Questionnaire (HAQ). The Dutch version of the HAQ comprises 11 items that are rated on a 5-point scale (completely disagree, disagree, neither agree nor disagree, agree, completely agree) [71]. Both a patient and a clinician version have been developed (example items include “I feel the clinician understands me”; “I understand the patient”). The HAQ contains two scales: Cooperation (Cronbach’s α = 0.88) and Helpfulness (Cronbach’s α = 0.76) [71]. Modest associations have been found between the therapeutic alliance and client outcomes in community mental health for patients with severe mental illness [72,73]. However, it has been noted that most studies performed in these settings have been limited by a lack of power and standardized measures [72]. Possibly, the current study can improve on these limitations.

#### Experienced stigma

Stigma will be measured using the 12-item ‘perceived devaluation and discrimination’ subscale of the self-report Stigma-Scale [74]. This subscale refers to the perception of common opinions about psychiatric patients, such as ‘Most people stay friends with someone who has had a mental illness’ and ‘Most people look down on people who have been hospitalized for mental illness’. The items are scored on a scale from 1 (strongly disagree) to 4 (strongly agree). A higher total scale score means more perceived stigmatization. The scale had acceptable reliability (Cronbach’s alpha = .78) and construct validity was demonstrated in studies predicting associations between stigma (as measured with the subscale of ‘perceived devaluation and discrimination’) and self-esteem, employment, demoralization, quality of life and treatment seeking in patients with mental illness [75,76].

#### Personality characteristics

The temperament dimensions from the Temperament and Character Inventory (TCI) [77,78] will be used to measure personality characteristics, in order to explore the relationship between temperament and motivation to engage in treatment. The temperament dimensions from Cloninger’s theory called novelty seeking, harm avoidance, persistence and reward dependence [78,79] are used in this study. Convergent validity exists in the form of studies comparing the TCI scales with other similar scales of validated personality tests [78]. The internal consistencies (i.e. Cronbach’s alphas) of the novelty seeking, harm avoidance, persistence and reward dependence subscales varied between α =0.62 and α =0.90 in psychiatric patients recruited from community mental health care [78]. The temperament dimensions are measured by items that can be scored as true or false.

### Procedures and randomization

**Figure 3:**
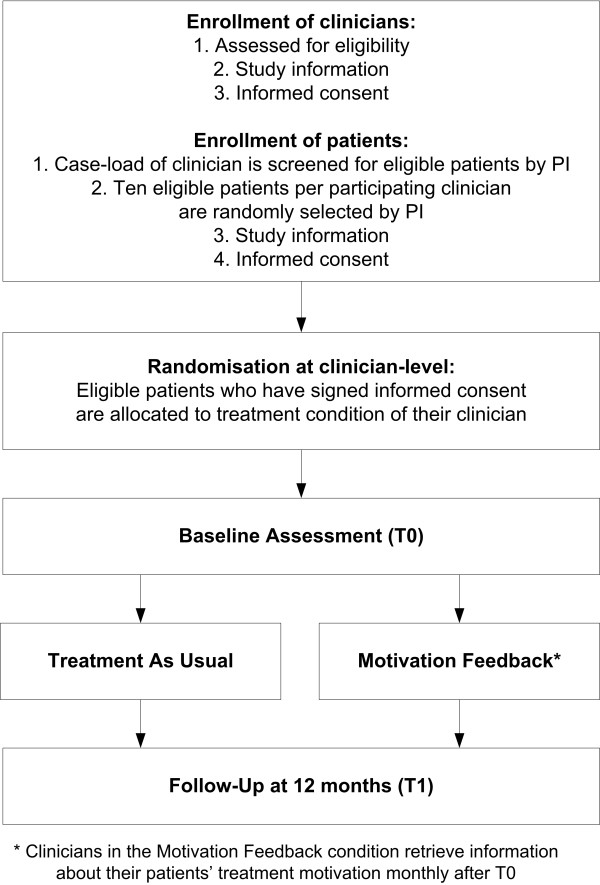
Flowchart of MotivaTe-IT procedures.

Figure 3 shows the study procedures. Eligible clinicians and patients will mainly be approached via specific treatment programs that provide FACT (for a description of FACT see the section on Treatment As Usual). Clinicians who are willing to cooperate in this study will be informed by the principal investigator regarding the goals and procedures of the study and receive an information brochure. Two weeks after having received the information brochure, clinicians will be contacted to ask for participation and to sign informed consent.

After having received informed consent from the clinicians, randomization will be performed at either clinician-level or team-level. Where clinicians work in FACT-teams, randomization will be performed at team-level so that a whole team (all clinicians working in this team) will be allocated to either the TAU condition or motivation feedback condition. As teams often work with a shared caseload between clinicians in the same team, this decision was made in order to prevent possible cross-over of the feedback-condition to the TAU condition within teams. Where clinicians work in an outpatient clinic on a one-to-one basis (individual case-management) then randomization will be performed at the clinician-level. The allocation ratio is 1:1 (i.e. therapist vs. therapist and team vs. team, respectively). Stratification for diagnosis in advance was considered unrealistic and impractical, as we would then have to achieve equal numbers of each patient diagnosis in each treatment condition, while our randomization is at team-level and clinician-level. Therefore, we chose to use multivariate modelling with diagnosis as a covariate (see section 2.9 ‘Statistical analyses’). Randomisation will be performed by assigning each randomization unit (e.g. a team or a clinician) a unique number, which is entered in a computerized randomization program (http://www.randomization.com) that randomizes each unit to a single treatment by using randomly permuted blocks. The randomization is single-blind, as both the principal investigator and clinicians need to know which condition the clinicians are in, in order for the clinicians to receive the necessary training for the intervention condition (or not). As a consequence, only patients will be blind to treatment allocation at baseline assessment, while clinicians are not. Due to the nature of the trial, follow-up assessment can not be blinded.

Subsequently, clinicians are asked to provide a list of their entire caseload to the principal investigator (PI). The PI will remove patients from this list who do not fulfil the inclusion criteria or fulfil the exclusion criteria and subsqequently, the PI will randomly select 10 eligible patients from this list to be asked for participation in the study. Clinicians will inform their selected patients about the objectives of the study, and provide a full explanation of all procedures for the study. If patients are willing to participate, an appointment is scheduled for the administration of the HoNOS.

At the beginning of the appointment, again all procedures of the research study are explained to the patient and signed informed consent will be obtained by the research assistant. Written information will also be provided to the patient, which explains the nature of the intervention and provides contact details of the research team. Following the informed consent procedure, baseline assessment will take place.

The HoNOS will be administered by the case-manager of the patient accompanied by an independent research assistant, who will assist in the interview and scoring of the HoNOS. This decision was made for several reasons. The first is that the case-managers have been trained to administer the HoNOS for Routine Outcome Monitoring, which is primarily used in clinical practice to guide treatment plans and evaluations and is now secondary used as an outcome in the current research study. Combining the two approaches ensures that Routine Outcome Monitoring procedures can be maintained (by the case-manager) while research requirements can be met (by the independent research assistant monitoring the administration and scoring of the HoNOS). Secondly, the response rate for the interviews is expected to be higher if the patient is approached by a familiar person (the case-manager). This might typically be the case for the more paranoid or anxious patients. Third, the presence of an independent research assistant who is also trained in the administration of the HoNOS likely ensures that the HoNOS is scored appropriately, to minimise a possible bias that might occur if the case-manager alone would do this. The self-report questionnaires will be administered by research assistants, only sometimes in the presence of the case-manager when the patient is seen at home to ensure the safety of the research assistant or to minimise feelings of anxiety with patients (who might feel intimidated by an unfamiliar person), but always ensuring the confidentiality and anonymity of the collected data.

Assessments of the HoNOS and self-report questionnaires will take place at baseline and follow-up at 12 months. Baseline assessment will take place after randomization to reduce the variation in the time between baseline assessment and the start of the intervention. Measuring baseline status close to the start of the intervention ensures that the information obtained at baseline assessment is still up to date at the start of the intervention. A limitation to this approach is that clinicians are aware of the treatment allocation, which may bias their responses. This possible information bias can not be eliminated since clinicians in the motivation feedback condition have to be trained in the relevant procedures before baseline assessment, since shortly after they will start employing the feedback intervention. Patients however, will not be informed about their treatment allocation at baseline assessment and are therefore blind to treatment allocation at the start of the study. In case patients drop-out from treatment or complete their treatment before these 12 months have passed, information regarding the reason for ending the treatment and total treatment duration will be obtained.

### Sample size and power calculations

The RCT was designed to enroll an average of 6 patients for each of 56 participating clinicians. The sample size was calculated on the basis of our primary hypothesis, that the intervention group (motivation feedback) would be more effective than the control group (treatment as usual) in enhancing treatment engagement, as measured with the Service Engagement Scale (primary outcome) at 12 months after baseline assessment. The difference between the motivation feedback group and control group for the primary outcome is based on a power of 0.80 and an alpha of 0.05 (two-tailed). Earlier studies regarding differences between feedback and treatment as usual (control) conditions have shown effect sizes (standardized mean differences) ranging from 0.34 to 0.92 [16,17], but were based on treatment progress and not (solely) on treatment motivation. One RCT studying the effects of treatment adherence therapy in patients with psychotic disorders used the SES as outcome measure and found an effect size of 0.39 [80]. Therefore, we expect an effect size of approximately 0.40. Using an unpaired t-test statistic, this resulted in a minimum of 123 subjects per condition. However, as patients are clustered within clinicians, and clinicians are clustered in teams, the patient and clinician observations can not be considered as independent of each other. The sample size was therefore adjusted by the (variance inflation) factor f = 1 + (m – 1)ρ, to account for the variance that would have been achieved had there been no clustering. The cluster size (m) is 6 (patients per clinician) and the within-cluster correlation (ρ) was estimated from a previous study to be around 0.07 [21]. Thus, the computed sample size was inflated by 1.35 to be at least 166 subjects per condition (minimally 332 in total). The SES is rated by clinicians and so we expect minimal loss to follow-up on the primary outcome, but to be on the safe side we will aim for 350 patients as the total sample size.

### Statistical analyses

The data of the RCT will be analyzed according to the intention-to-treat principle. Baseline comparability between the intervention group and control group in demographic and clinical variables will be evaluated with independent samples t-tests and chi-square tests. Furthermore, non-responders (i.e. eligible patients who chose not to participate in the study) will be compared to responders with respect to background demographic and clinical variables with independent samples t-tests and chi-square tests. Logistic regression analysis will be applied to test for differences between the motivation feedback and control group with respect to the primary and secondary outcomes that are dichotomous variables, while (multiple) linear regression analysis will be used in case of continuous outcome variables. For individual categorical outcome variables, the effectiveness will be determined by odds ratios, including p-values (two-tailed). The effectiveness of the variables combined will be determined by ROC-curves (for categorical outcomes) and the individual odds ratios, R2 and the individual regression coefficients (for continuous outcomes). The Hosmer and Lemeshow goodness-of-fit test will be used in case of logistic regression. In case of multiple regression analysis the classical regression diagnostics will be applied for normality, (non)linearity, heteroscedasticity, (influential) outliers and interaction. A subgroup analysis will be performed for patients with psychotic disorders for the effects of the intervention upon their antipsychotic medication adherence. The analyses will be performed both unadjusted and adjusted for baseline differences of the distributions between the two treatment groups. In analyzing a specific outcome variable, the baseline score of that variable will be used as covariate. The analysis will be extended using multilevel analyses that takes the nesting of measurements into account. A clustering of outcomes is likely since a single clinician may treat several patients, and clinicians are clustered into teams. Multilevel modelling will be performed to check for any clustering effects on the primary outcome. In the multilevel analyses we consider the two measurements as the first level and the patient as the second level. We will explore whether the different treatment locations (FACT teams) and institutions (MHC Breburg and MHC WNB) can be considered as random factors in the modeling. We will identify predictive factors in estimating the outcome and whether there are predictive factors dependent on the type of treatment condition (interaction between baseline variables and treatment effect). Furthermore, we will take into account to what extent patients were exposed to the intervention by analyzing the dose-effect relationship. We expect (as is the case in most empirical studies in a psychiatric setting) that missing data will occur. We expect that the data will be Missing At Random (MAR), which is allowed to be a function of the observed variables (both covariates and outcome variables). If the assumption of MAR is violated, the pattern mixture model approach will be applied. In case predictor variables are missing, the method of multiple imputations or the maximum likelihood estimation method will be applied.

For monthly measurements (i.e. the motivation feedback graph for patient and therapist, and the therapist motivation) the method of mixed modelling will be applied. This highly flexible method enables two level models: repeated measurements (level 1) and patient level (level 2).

The three motivational theories will be modelled with Structural Equation Modelling (SEM), in order to study their fit to the empirical data, their predictive power and parsimony (i.e. whether the model can be simplified without substantially reducing the model fit and predictive power). The three motivational theories will be studied exploratively to determine which theoretical constructs are most plausible (i.e. clinically relevant and statistically significant) for the prediction of the outcome variables. The difference of the two -2log-likelihood tests (including the difference of degrees of freedom) will be used for testing differences between nested models, and information criteria will be used for differences between non-nested models (i.e. Akaike Information Criterion/AIC, Bayesian Information Criterion/BIC and adapted BIC). Where relevant, the 95% confidence intervals and/or P-values (two-tailed) will be reported.

### Ethical considerations

The current research protocol was endorsed by the Medical Ethical Committee for Mental Health Care Institutions (METiGG) and by the committees for scientific research within the two mental health institutions where the data will be collected (MHC WNB and MHC Breburg). The collected data are treated according to the Medical Confidentiality Rules, and are kept in locked files cabinets. Every patient will be assigned a patient number, so that processing of the data will occur anonymously. Access to data is limited to members of the research group and the medical ethical committee (METiGG). The study will be conducted in accordance with the Helsinki Declaration. As mentioned previously, written informed consent will be obtained for all clinicians and patients that are entered into the study. Patients and clinicians are free to refuse participation at any time during the research period, without having to disclose any reason why.

Patients that are included in the study will receive an incentive of € 15, - after every completion of an extensive measurement (baseline and follow-up). Thus, if a patient has completed both measurement occasions, he or she will have received € 30, - in appreciation of his/her cooperation. These incentives are introduced in order to increase the response rate, since it is expected that in this patient population with severe mental illness and possibly with motivational problems, the response rate would otherwise turn out too low. The effects of the intervention are unknown at this moment, and therefore we think it is justified to allocate patients randomly over the two conditions.

## Discussion

The central research question in this study is whether the motivation feedback intervention is able to increase the treatment engagement of patients in outpatient psychiatric treatment for severe mental illness. The secondary research question is whether the intervention improves treatment motivation, psychosocial functioning (health and functioning in several life domains) and quality of life. Thirdly, three theories of motivation will be assessed on their core theoretical constructs to investigate which theoretical constructs and which theory is best able to predict the outcomes in this patient population. The identification of possible mediating and moderating mechanisms through which changes in the outcomes occur, offer a tool for the development of future interventions. The study has several strengths and limitations.

### Limitations

The main limitation of the design is that patients and clinicians are not blind for the treatment condition to which they are randomized. Clinicians will be informed about their treatment condition, since it is required that clinicians in the motivation feedback condition receive training. Patients are blind for treatment condition at the baseline assessment, but not at follow-up assessment since they will realize what condition they are in once their clinician starts asking them to fill in the feedback questionnaires monthly after baseline assessment – or not. This could lead to information bias, as patients and clinicians in the intervention group may be more actively involved in the treatment as they expect it to work, which may enhance the effect of the intervention we find. This would especially be the case for the subjective (i.e. self-report) outcome measures that are administered to patients and clinicians, but less so for the objective outcome measures (e.g. number of no shows and drop-out as registered by the institution’s administrative system). Regarding the HoNOS, which is administered by the patient’s case-manager and an independent research assistant, we have weighed the possible bias that could occur due to the presence of the case-manager with the advantage of achieving higher response rates for the study, thereby minimising a possible selection bias (that would occur if the more severe mentally ill group would decline participation if asked by an unfamiliar person). We believe that the presence of the independent research assistant during the administration and scoring of the HoNOS ensures that the HoNOS is scored appropriately and will minimise the former bias. A second limitation is that it is not possible to determine which exact component of motivation feedback contributed to the effect, since it might be possible that measuring patient progress systematically in itself is key to the effects – whether you measure the patient’s motivation or the patient’s symptoms or any other patient characteristic – or the fact that the intervention includes reminders to the clinician to keep in contact with the patient for the measurement of the motivation. In order to have some idea of which elements contributed to the effect of the intervention, we will monitor the number of times the feedback was used, the amount of time that was spent on discussing the feedback, characteristics of clinicians using the feedback and the motivation of the clinician to treat the patient. Thirdly, the DSM-IV diagnosis is not established with structured diagnostic interviews, but is obtained from the patients’ medical records. This choice was made to reduce patient burden, since structured interviews were considered too extensive and time-consuming in combination with the other instruments used in this study.

### Strengths

The strengths of this study include the design and the clinical relevance. The patients in the study are retrieved from a general population of severe mental illness (i.e. psychotic disorders and personality disorders), representing a ‘real-life’ population including patients with a variety of comorbid disorders rather than a more narrow selection of patients. Therefore we will be able to generalize our findings to a large group of outpatients with psychotic disorders and personality disorders. The design of the motivation feedback intervention is based upon empirical evidence of interventions that have proven efficacious in lowering treatment non-completion and drop-out. Most of the studies concerning feedback have been based upon self-report measures from the perspective of the patients. The current study also incorporates the clinicians’ perspective upon the patients’ motivation for treatment. Also, past research concerning the effects of feedback has largely included patients with relatively mild problems and non-specific disorders (for example, the studies by Lambert et al. [13,14] were based on data from a university outpatient clinic). The current study will focus upon patients with severe psychiatric problems.

Regarding the theory comparisons it should be noted that SDT will be tested most rigorously in this study, since this theory will be used as the basis for the intervention in this study and its core theoretical components will be manipulated (i.e. the basic psychological needs will be supported by clinicians, and motivational types will be known and responded to by clinicians). Although the other two theories are not tested so rigorously (i.e. they are not part of the intervention), the core theoretical constructs of IM and TTM are followed prospectively over the course of 12 months in order to determine if the constructs behave as the theories suggest and to see if they are able to predict treatment motivation and treatment engagement at follow-up. The design of our study fulfils most of the criteria that have been suggested by Noar and Zimmerman [81] for theory comparison studies. The criteria are: 1) having a longitudinal design, 2) using Structural Equation Modelling, 3) including past behaviour and (4) demographics in the model tests, 5) including non-college participants in the sample, 6) having a strong sample size (N>200), 7) utilizing multiple samples in model testing, 8) utilizing samples from more than one country, 9) having more than one dependent variable (e.g. motivation and behaviour), 10) examining more than one behaviour, 11) comparing more than two theories and (12) empirically examining an integrated model [81]. All criteria except 8 and 10 are fulfilled by our design. Furthermore, most previous studies employing the TransTheoretical Model have only measured the stages of change, while the model also incorporates other constructs. The current study measures both the stages of change, the processes of change, self-efficacy and the decisional balance constructs. Thus, a strong aspect of this study is that it includes all core theoretical constructs of the three motivational theories.

## Competing interests

The authors declare that they have no competing interests.

## Authors’ contributions

ECJ wrote this manuscript and is the principal investigator of this study. ECJ, CLM, AD and HJD designed the study. CLM leads the project and CFC, AD, HJD and SCMS supervise the project. HJD is the statistical advisor of the project. CLM, CFC, AD and HJD helped to draft the manuscript and provided comments. WS is a contributing researcher in this study and will focus specifically on the medication adherence in patients with a psychotic disorder. SCMS is also a contributing researcher in this study and will focus upon the level of agreement between clinicians and patients regarding their motivation for treatment. All authors read and approved the final manuscript.

## Pre-publication history

The pre-publication history for this paper can be accessed here:

http://www.biomedcentral.com/1471-244X/12/209/prepub
